# Interpreting outcome following foot surgery in people with rheumatoid arthritis

**DOI:** 10.1186/s13047-016-0153-6

**Published:** 2016-07-08

**Authors:** Michael R. Backhouse, Karen A. Vinall-Collier, Anthony C. Redmond, Philip S. Helliwell, Anne-Maree Keenan

**Affiliations:** Leeds Institute of Rheumatic and Musculoskeletal Medicine, University of Leeds, 2nd Floor Chapel Allerton Hospital, Harehills Lane, Leeds, LS7 4SA UK; NIHR Leeds Musculoskeletal Biomedical Research Unit, Leeds Teaching Hospitals NHS Trust, Leeds, UK; Dental Public Health, University of Leeds, Leeds, UK; Academic Unit of Health Economics, University of Leeds, Leeds, UK; Bradford Teaching Hospitals NHS Foundation Trust, Bradford, UK; School of Healthcare, University of Leeds, Leeds, UK

**Keywords:** Rheumatoid arthritis, Foot surgery, Outcome, Surgeon, Footwear, Activity, Participation

## Abstract

**Background:**

Foot surgery is common in RA but the current lack of understanding of how patients interpret outcomes inhibits evaluation of procedures in clinical and research settings. This study aimed to explore which factors are important to people with RA when they evaluate the outcome of foot and ankle surgery.

**Methods and Results:**

Semi structured interviews with 11 RA participants who had mixed experiences of foot surgery were conducted and analysed using thematic analysis. Responses showed that while participants interpreted surgical outcome in respect to a multitude of factors, five major themes emerged: functional ability, participation, appearance of feet and footwear, surgeons’ opinion, and pain. Participants interpreted levels of physical function in light of other aspects of their disease, reflecting on relative change from their preoperative state more than absolute levels of ability. Appearance was important to almost all participants: physical appearance, foot shape, and footwear were closely interlinked, yet participants saw these as distinct concepts and frequently entered into a defensive repertoire, feeling the need to justify that their perception of outcome was not about cosmesis.

Surgeons’ post-operative evaluation of the procedure was highly influential and made a lasting impression, irrespective of how the outcome compared to the participants’ initial goals. Whilst pain was important to almost all participants, it had the greatest impact upon them when it interfered with their ability to undertake valued activities.

**Conclusions:**

People with RA interpret the outcome of foot surgery using multiple interrelated factors, particularly functional ability, appearance and surgeons’ appraisal of the procedure. While pain was often noted, this appeared less important than anticipated. These factors can help clinicians in discussing surgical options in patients.

**Electronic supplementary material:**

The online version of this article (doi:10.1186/s13047-016-0153-6) contains supplementary material, which is available to authorized users.

## Background

The propensity of rheumatoid arthritis (RA) to affect the joints of the feet is well established and foot pathology is thought to be almost ubiquitous in people with RA [1]. Many patients eventually require foot surgery and although there is a wide array of surgical procedures available, outcomes are mixed and often sub optimal [2–4]. Furthermore, while surgery is episodic patients will often remain under the long-term management of a clinical team following foot surgery. It is therefore important for these teams to be aware of the complexities of how patients report surgical outcomes, as greater understanding will aid clinicians’ own evaluation of surgical outcomes and help inform future treatment decisions.

The importance of a patient-centered approach is now widely regarded as a crucial component in the delivery of high quality care and has led to recognition of the key role of the patients when determining treatment outcomes [5, 6]. This fundamental lack of understanding of what is important to people with RA having foot surgery not only hampers the research required to refine surgical procedures and reduce variation in outcome but also hampers clinical consultations [7]. Truly patient centred care requires clinicians to delve beyond standardised PROMs, which are developed on a group level, and explore issues that are important to the individual patient during consultations.

Therefore, the aim of this study was to explore which factors are important to people with RA when they evaluate the outcome of foot and ankle surgery.

## Methods

### Participants

A convenience sample of patients with RA who had previously undergone foot surgery was recruited over a 7 month period from the rheumatology outpatient departments at two local hospitals which serve wide geographical areas covering urban and rural areas, with wide social and ethnic variance. Patients were eligible if they had a primary diagnosis of RA and had undergone elective foot or ankle surgery. Patients were not approached on the basis of age, gender, or surgical outcome. Surgery was conducted in four NHS Trusts and one private clinic. Patients were approached by members of their rheumatology team, provided with a patient information sheet, and those who assented were contacted to discuss the details of the study. Only one patient declined to participate: no reason was provided. All participants provided written consent prior to starting the interviews.

Full NHS ethical approval was obtained from Bradford REC and all participants provided written informed consent (Ref 08/H1302/2).

### Data collection

The data collection method used most closely allies itself to a phenomological philosophical qualitative methodology. Phenomenology is a school of thought that emphasizes a focus on people's subjective experiences and interpretations of the world and is particularly useful when attempting to understand how the world appears from the lived experience.. Thematic analysis is a method of organising and structuring themes in order to gain an understanding into the comprehension or meaning of a concept [8]. Thematic analysis is commonly used in phenomenological approaches to data analysis and is considered a structured method of exploring themes through a conceptual matrix [8]. In essence, it is a method of bringing together componetns or fragments of an idea that relate, which are often meaningless when viewed alone, but form a comprehensive picture of the collective experience [9].

In order to collect this “lived experience”, in-depth semi-structured interviews were performed by the researcher (MRB), a podiatrist,. Following one on one training, MRB was shadowed by AMK for the first three interviews, where structured feedback was provided. The interviews were conducted with the aid of an interview guide (Table 1) which was developed following narrative review of the literature and informal discussions with patients as to what was important to them in their surgery: the guide was reviewed by the research team after the first, third, and fifth interviews. Free conversation was encouraged and no time constraints were placed on the interviews in order to allow free and open exploration of the issues.

**Table 1:** Interview topic guide

a) Can you start by telling me a bit about your arthritis?
b) Tell me about the pain in your feet and how it effects you?
c) Can you tell me about the operation you had on your foot?
d) What was important to you when you decided to have the operation?
e) Was the operation successful in your opinion?
f) Did you do anything that you did effected the outcome of the operation, or do you think the success/failure of the operation was all down to the surgeon?
g) What was the worst part of having the operation?
h) Did anything about the operation surprise you?
i) What do you think having the operation on your foot has changed?
j) What things are important to you when you are deciding whether the operation on your foot was successful?
k) Knowing what you know now, would you have the operation again?
l) Do you have anything else you would like to mention that we haven’t discussed?

Interviews were audio-recorded and transcribed verbatim by a third party, but with names and identifiable details removed to ensure anonymity of both the participant and their healthcare professionals. Accuracy of the transcripts were verified by the researcher (MRB) against the audio recording but were not verified by participants. Field notes were made by the interviewer (MRB) to help record non-verbal cues and add contextual detail to help with subsequent analysis.

### Analysis

For the purposes of this exploration of the data, thematic analysis focussed primarily on themes identified at the semantic or explicit level. The analysis then progressed from a description of patterns in the content to offer some interpretation in an attempt to theorise the significance of the patterns and their broader meanings, implications and in relation to previous literature [10].

An inductive or ‘bottom up’ [11] approach was adopted meaning that the themes identified are strongly linked to the data themselves [10]. The researcher did not try to fit to a coding frame or take into account previous work in the area in an attempt to get a ‘data-driven’ analysis of themes.

Interview transcripts were analysed thematically according to the principles outlined by Braun & Clarke [12]. After a period of familiarisation through immersion in the data by reading and reviewing the data on several occasions, an initial coding scheme was developed by members of the research team. All interviews were then coded using NVIVO 8 (QSR International, Doncaster, Victoria, Australia), with new ‘data driven’ codes emerging during the analysis. This iterative process was repeated until no new codes emerged from the data.

The initial approach to coding was determined by the research team, who included two podiatrists (MRB and AMK), and a psychologist (KVC), two of whom have experience in thematic analysis (AMK and KVC). MRB initially coded all transcripts with AMK and KVC cross checking and recoding six of the transcripts. Any disagreements were identified and discussed until consensus was reached.

**Fig. 1:**
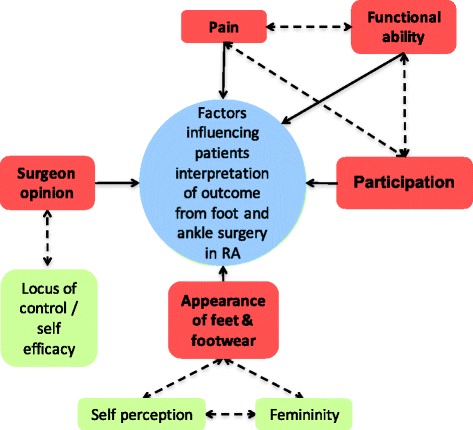
Interrelationship of themes used by patients when evaluating surgical outcome. The major themes are highlighted in red. Dashed lines indicate interrelationships between themes

After review of the coding, the initial coding structure was reviewed by the research team. This phase refocused the analysis to the broader level of themes rather than individual codes and attempted to identify broader overarching themes that were emerging from the data. The interviews were then reviewed and themes were revised until the research team agreed they adequately captured the complexity of the data. Further details of the process of thematic selection are presented in Additional file 1: Table S1. The research team (MRB, KAV, AMK) then undertook a final review of consistency in interpretation of themes. Quotations representing each of the coding clusters were taken from the transcripts and KAV and AMK coded the statements using the second generation themes. Full transcripts of the interviews were available if the coders felt they needed further information the context of the quote although this was not requested. Finally, themes were drawn together in a thematic map (Fig. 1) to highlight the interrelationship between themes and illustrate the major contributing factors of how patients determine the outcome of their operation.

## Results

Eleven participants were interviewed but on review of the transcripts, the research team considered that the interview of one patient was to be excluded due to the excessive influence of her niece on the conversation. Therefore interviews of ten participants are included in the analysis. Participant characteristics are described in Table 2.

**Table 2:** Demographic characteristics of participants. All numerical variables are provided in years

Patient ID	Gender	Age	Disease duration	Time post op	Location of surgery
Participant 1	Female	68	14	6	Forefoot
Participant 2	Female	69	12	2	Forefoot
Participant 3	Female	61	12	3	Forefoot
Participant 4	Male	33	6	1	Rearfoot
Participant 5	Female	71	11	2	Forefoot
Participant 6	Female	81	20	4	Forefoot
Participant 7	Male	81	30	3	Forefoot
Participant 8	Female	58	21	2	Forefoot
Participant 9	Female	34	12	1	Forefoot & Rearfoot
Participant 10	Female	54	25	2	Forefoot

Participants’ reasons for having the surgery varied and were often multiple, these included pain, impaired walking and limitations in footwear choices. Three participants had undergone the surgical procedure based primarily on the opinion of healthcare professionals including podiatrists and rheumatologists. Four participants were concerned about preventing further damage in other joints, both within the foot and elsewhere in their body. Participants often reported several surgical procedures, on several different joints, and often for numerous reasons.

Participants identified a multitude of factors associated with their experience of foot surgery as a patient with RA. They differed in what they considered to be important with regard to the outcome of their operation and in their explanations of what influenced these factors. Despite a number of participants reporting problems following surgery, including continued pain and the need for revision surgery, they reported high levels of overall satisfaction. Five major themes emerged from the data which were strongly associated with the participants’ views of the outcome of their surgery: functional ability, participation, appearance of foot, appearance of footwear, surgeons’ opinion, and pain. These are highlighted in Fig. 1.

One interesting finding was the level of overall satisfaction: five out of the ten participants reported their surgery to be a success, with another four saying it was a qualified success. Only one of the participants deemed the outcome as negative, in that the shape of their foot was made worse, which made footwear choices even more limited, resulting in additional pressure on the joint prominence and subsequent long term ulceration. Eight of the ten participants indicated they would have the operation again, reflecting their positive perception of outcome.

### Theme 1: Functional ability

The ability to undertake key physical activities was considered important to participants, with several reporting that improved walking ability was a key surgical outcome. Unsurprisingly, the participants’ expectations of activity levels were often tempered by their overall expectations in the context of their wider disease and their functional ability prior to their surgery:

*“You know you can't walk as far and be comfortable and with your feet being uneven underneath you always need to hold on to someone, you know before I had my feet done, which is not as bad now..... with me having rheumatoid arthritis my hands are the very worst part of me so that affects a lot of things I can do.”*

*Participant 3, F, 61 years*

Interestingly, positive feelings of improved mobility were often moderated by negative self-perception, which was often related to the effect of RA on their appearance:

*“Definitely, cos it’s [the surgery] taken..... the worse pain away..... I mean I still walk like a waddling duck”*

*Participant 2, F, 69 years*

### Theme 2: Participation

While functional ability was considered important, this had the most impact as a surgical outcome when participants were able to engage in valued activities following surgery. Although many of the themes are interrelated, the relationship between functional ability and participation in valued activities was probably the closest as walking is in itself a valued activity to many participants.

*“one thing we do love doing is you know to take the dogs off up to the Dales or go for a weekend somewhere and go walking so I mean we don’t hike we don’t go for twenty miles or anything but just a pleasant walk and so that was one of the reasons that pushed me into doing it [the surgery] really.”*

*Participant 10, F, 54 years*

It was not just being able to undertake physical activity that they valued but also fulfilling their role within the family and society:

*“that affects a lot of things I can do with the grandchildren and if you're in pain you just can't do anything really, you know but when you're pain free you're OK, but like with your feet I was limited to what I could do but now I can do that bit more with them it is better.”*

*Participant 3, F, 61 year*

### Theme 3: Appearance of feet and footwear

One of the most important and complex issues identified was the impact that surgery had on physical appearance: almost all participants thought that this was important. Participants reported embarrassment about the pre-surgical physical appearance of their foot.

*Well just the looks of your feet really you know, just the looks of the feet, they were awful they really...... they look a lot better than they did and you know I never liked to take my shoes off before or my socks of or anything which you know I don’t mind now sort of thing.*

*Participant 3, F, 61 years*

While the change in the appearance of the foot was considered important, participants clearly felt the need to legitimise this as an outcome and frequently entered into a defensive repertoire:

*“it’s not cosmetic surgery, it’s something more important than that, you know”*

*Participant 9, F, 34 years*

Participants were not only concerned with the appearance of their foot, but also the appearance of their footwear. Limited choice in footwear was a common source of pre-operative distress to many participants and was seen as both a key motivator for surgery:

*“If you hadn’t got footwear that looks what you consider, not necessarily right up to date but normal, feminine it’s you know doesn’t matter if you’re wearing couture, it doesn’t matter. If your footwear’s not right well you just don’t look good to yourself.”*

*Participant 1, F, 68 years*

and an important outcome, although it was modified by the context of their disease:

*“....obviously they [shoes] look better but erm I don’t have to bother about dancing shoes any more so you know it’s still important that you want to look normal”*

*Participant 1, F, 68 years*

### Theme 4: Pain

Whilst pain was important to almost all participants, it appeared to be less important than the other themes. Pain was predominantly raised as an issue when it influenced other themes such as participation or physical activity. Of note, one participant felt the need to legitimise their pre-surgical foot pain in order for health professionals to take it seriously:

*“in the end I went to my GP because it had happened a few times and I went to an orthopaedic surgeon … who was quite dismissive of it, it was like what are you complaining about, type thing, which was a bit upsetting”*

*Participant 10, F, 54 years*

Despite participants viewing the outcome of their surgery as successful, many reported continued pain and discomfort in their feet, which was sometimes, but not exclusively, linked with their goals for surgery. Participants reported ongoing problems at the site of their operation, as well as more widespread foot involvement and new problems following surgery:

*“I still have some discomfort as I say because of corns and this and that”*

*Participant 7, M, 81 years*

When talking about the effect of surgery on their pain, participants described relative changes in their pain rather than in terms of whether or not the pain had completely disappeared:

*“No, no, it aches, it aches but it's not that no, that grind.”*

*Participant 2, F, 69 years*

### Theme 5: Surgeons’ opinion

The opinion of the surgeon was clearly important to participants both in the decision of whether to have the procedure and the outcome of surgery.

*“I thought well if I'm going to pay this much money I should listen to the surgeon so I just put my faith in him”*

*Participant 9, F, 34 years*

*“The fact that he was pleased meant that it was alright and it had been successful.”*

*Participant 9, F, 34 years*

This was evident, even when the surgeons’ opinion differed to that of the patients’ current opinion and their initial rationale for having the surgery.

*“when he’d done it the first time he saw me he said that hasn’t worked as good as he’d wanted to… but the pain has gone”*

*Participant 2, F, 69 years*

## Discussion

Interpretation of the outcome of foot and ankle surgeries in people with RA is multi-faceted and interrelated. This study identified that a complex array of factors influence patients’ interpretation of outcome and that the importance of these factors varies between patients and in relation to the patients’ life context. Several themes were identified as important with functional ability, participation in valued activities, pain, and the surgeons’ appraisal of the outcome appearing to have the largest influence on how patients interpret their outcome.

Participants in the current study were predominantly older females who had lived with RA for many years, which is typical of those undergoing such surgery [13]. All reported a history of labile disease activity, with many recounting acute episodes of flare and multiple failed medications. Clearly the impact of such a chronic and disabling disease and its ongoing management had an impact on participants’ experience of surgery as well as their interpretation of outcome. While it was clear that the impact of their RA and foot pathology has a considerable impact on most aspects of their daily lives, the majority of participants had appeared to have developed a stoical approach to their RA and were initially reluctant to dwell on the negative impact it had on their lives. Previous research suggests that this approach is intimately linked to self-efficacy, the belief that one can control one’s response to their health condition, and has been found to be associated with positive treatment outcomes [14]. Importantly higher self-efficacy has been identified as a coping mechanism for living with chronic illness in general [15] and RA in particular [16]. Self-efficacy has also been identified as a predictor of postoperative functional ability in orthopaedic surgery [17, 18].

One of the most intriguing findings in the current study was that satisfaction was high, despite many participants reporting continued pain and significant complications, including revision surgery. Although there may be an element of the Hawthorne effect here, this is the first time this discordance has been reported in relation to foot surgery. Similar findings have been reported previously in patients following total knee replacement [19]. Woolhead et al. [20] reported that when asked a direct question as to how satisfied patients were with their knee replacement, the majority provided a positive response, which the authors described as a public or ‘socially desirable’ positive summary of their operation. These responses changed to reveal a different ‘private expression’ after further in depth questioning. Such public expressions of satisfaction are complex: they may be influenced by a patient’s desire to justify their decision to go ahead with the operation; or to express gratitude that the operation had been performed and that something had been done to relieve some of the pain they were experiencing often after several years of waiting. Indeed, the complex interaction between gratitude and satisfaction is difficult to unpick: interestingly, this has been reported as an issue in long-term conditions, particularly rheumatoid arthritis [21].

Patient satisfaction is a widely used, but poorly defined concept in healthcare and although definitions vary, they generally centre on satisfaction being the extent to which an individual’s experience meets their expectations [22–24]. Patient expectations are not a stable trait over time and studies have shown that patient expectations can be deliberately modified, and that surgeons frequently guide patient expectations towards what they consider to be achievable [25, 26]. Modification of patients’ expectations would in turn influence their final level of satisfaction.

The high levels of satisfaction in the presence of ongoing pain and complications may also be associated with the concept of a “response shift” whereby patients re-evaluate the values and activities that give their life meaning following a therapeutic intervention [27, 28]. Certainly, evidence for a response shift has been identified in other types of orthopaedic surgery [20]. Whatever the reasoning behind patients’ thought processes, the importance and influence of the surgeons’ opinion to participants was a clear finding of the current study.

Notable amongst the interviewees was the high regard in which the participants held their surgeons and the absence of blame apportioned to the surgeon for any post-operative complications or other ongoing problems they experienced. The high regard in which the surgeons were held permeated beyond an unwillingness to blame them for negative aspects of their outcome, to a willingness to have revision surgery at the suggestion of the surgeon, even in one case when the operation had achieved the patient’s initial goals. Whilst this may in part be due to the persistence of a paternalistic model of patient-clinician care, where patients defer to the clinicians greater knowledge, independent of their own values or desires [29], it may also reflect the wider societal view of surgeons, with one patient referring to “the hands of the surgeon God” (Participant 9). Indeed, the importance of the symbolic power the surgeon and the prestige that this deemed to confer is one that has received considerable attention [30].

The influence of the surgeons’ own appraisal of surgical outcome has on the patients’ own interpretation was surprising and, to our knowledge, has not been reported previously. However, this study relies upon patient reporting only and so is subject to recall and interpretation bias: patient understanding may not necessarily be the same as the surgeons’ appraisal and future work could investigate how surgeons communicate surgical results to patients and the impact this has.

Pain and functional impairment emerged themes closely related in the interviews. It was noticeable that when participants talked about their current levels of pain and physical impairment, that they discussed them in terms of change in relation to their preoperative levels and in the context of their wider disease. This is again evidence of the positive coping strategies demonstrated by people with RA [16]. As participants were more concerned about the magnitude of change in pain and functional ability associated with their surgery, rather than the absolute levels, this has important implications for the interpretation of outcomes in future research.

When participants discussed the role of pain in determining their outcome, they always did so in conjunction with the other themes, and particularly how pain influenced their ability to participate in valued activities: they saw the effects of pain to be more important than the level of pain itself. This is consistent with findings reported in the wider musculoskeletal literature and is often referred to as “illness intrusion”, whereby it is not the physical symptoms that are necessarily important to patients, but the impact of them on the person’s ability to perform valued tasks and activities [31, 32].

Appearance was frequently mentioned by participants and consisted of two interrelated aspects; the appearance of their foot, and the appearance of their footwear. The psychological impact patients experience when they are limited to therapeutic footwear is well documented, and there is now evidence that commercially available footwear causes similar issues [33, 34]. Patients, particularly women, report that wearing therapeutic footwear causes feelings of shame, sadness, anger, social isolation, and loss of choice and femininity [33, 35, 36]. Findings in the current study reiterate the impact caused by footwear but also highlight that footwear is also a motivating factor for surgery and should be included when measuring surgical outcomes in future studies.

The appearance of the foot itself was also a source great embarrassment for some participants, although less so than footwear. Whenever participants admitted that the appearance of their foot was important, it was accompanied by strong feelings of needing to emphasise such concerns. Similar findings have been found in studies of patients with RA undergoing hand surgery where hand appearance has been identified as a major motivator for surgery and determinant of satisfaction [37–39]. A patient’s hands are generally uncovered however and any deformity is exposed to others, particularly when shaking hands. This is not the case with a person’s feet as these are normally covered by footwear which must accommodate such deformity, so it is the appearance of accommodative footwear that people seem to be more embarrassed by rather than the appearance of the foot itself [33].

There is a possibility that the high levels of patient satisfaction reported in the current study, may limit the ability to capture other pertinent factors. Patient satisfaction is a limited indicator of surgical outcome particularly given the complex interaction with patient gratitude. It could be that dissatisfied patients use alternative factors to determine the outcome of their surgery so these may not be fully captured in in the current study. Although this remains a possibility, there was no evidence to support this notion in the current interviews, and the major themes identified here appear to be important to both satisfied and dissatisfied patients.

While this study is the first to explore the themes relating to patient’s experiences following foot and ankle surgery in RA, we acknowledge several limitations. The small number of participants is consistent with the methodological approach employed in this study, but this limits the generalisability of findings [40]. Further research should seek to determine the extent to which these findings are replicated in larger groups of people with RA who have undergone foot surgery. The sampling technique used in the current study was that of convenience which meant that patients were not sampled purposively and this may influence thematic selection, particularly with a high proportion of female participants. Offsetting this, participants were recruited from two centres and have similar proportion of females to previous studies of foot and ankle surgery which recruited people with RA across a much wider a geographic area [41]. It is not clear however, whether provision of surgical care in these regions is representative of the wider national picture. Finally, as participants had already undergone surgery it was not possible to explore fully their preoperative goals or expectations and how these related to subsequent overall satisfaction and outcomes. It is certainly possible that recall bias effected participants’ recollection of what motivated them to have their operation at the time. However, even if this were an issue, it would not detract from the validity of their expression of which factors were important to them at the time of interview. Future prospective research to investigate this is warranted.

## Conclusion

In conclusion, patients interpret the outcome of foot and ankle surgery using a multitude of interrelated factors which should be incorporated into future research and clinical practice. Clinicians should be aware that reported satisfaction with surgery may be high despite patients experiencing continued problems following surgery, and so must not rely on superficial responses when evaluating surgical outcomes. Instead, questioning should explicitly explore the themes identified here: functional ability; appearance of the foot itself and the impact of wearing undesirable footwear; and surgeons’ opinion of outcome were paramount. While persisting pain was frequently noted, this was generally considered less important than other factors in the overall perception of surgical outcome.

## Abbreviations

NIHR, National Institute for Health Research; PROM, Patient Reported Outcome Measure; RA, rheumatoid arthritis

## Additional file

Additional file 1: Table S1. Evolution of codes used in thematic analysis. (DOC 80 kb)
