# Supplementary material for: Interpreting outcome following foot surgery in people with rheumatoid arthritis
Source: J Foot Ankle Res. 2016 Jul 8;9:20. doi: 10.1186/s13047-016-0153-6 (PMC4938997; doi:10.1186/s13047-016-0153-6)
Supplement: Additional file 1: Table S1. — Evolution of codes used in thematic analysis. (DOC 80 kb) [file 13047_2016_153_MOESM1_ESM.doc]

**Additional file 1: Table S1**

Evolution of codes used in thematic analysis

| **Initial Coding** | **Coding Clusters** | **Second Generation Themes** |
| --- | --- | --- |
| Impact of foot pain on activities | Activity limitation |  |
| Occupational impact of foot pathology |  | Frustration at impact of feet on activities |
| Participation in valued activities |  |  |
| Concerned about appearance |  | Participation limitation |
| Change in appearance Pre & Post Op |  |  |
| Comparison with other sites in body |  |  |
|  |  |  |
| Cosmesis Vs necessity | Shape and appearance of foot |  |
| Deformity |  |  |
| Not wanting to Look like an old person |  |  |
| Shoes Vs Feet |  |  |
| Ugly shoes |  |  |
| Wanting to look normal |  |  |
|  |  |  |
| Appearance of footwear |  |  |
| Custom Footwear |  |  |
| Femininity | Footwear |  |
| Limited choice of footwear | Femininity |  |
| Desire to wear 'normal' shoes |  |  |
|  |  |  |
| Importance of surgery | Interaction with other |  |
| Advice from other health professionals |  | Opinions of other health professionals |
| Effectiveness of conservative therapy |  |  |
|  | legitimisation of pain |  |
| External locus of Control | Locus of control / self efficacy |  |
| Internal locus of Control |  |  |
|  |  |  |
| Post operative mobility issues |  |  |
| Healing complications |  |  |
| Time spent in hospital | Negative post op experiences | Outcome - negative |
| Post Operative pain | Post op complication |  |
| Metalwork removal |  |  |
|  |  |  |
| Pre RA foot problems | Other foot problems |  |
| Non RA foot problems | Pre op ulcer |  |
|  |  |  |
| Other people's perceptions of footwear |  |  |
| Other people's perception of RA in general | Other people's perception |  |
| Other people's perception of importance of surgery | Self perception |  |
|  |  |  |
|  |  |  |
| **Initial Coding** | **Coding Clusters** | **Second Generation Themes** |
| Importance of activities to outcome | Outcome |  |
| Importance of appearance to outcome |  | Outcome - positive |
| Importance of footwear to outcome |  |  |
| Importance of pain to outcome |  |  |
| Importance of pressure post operatively |  | Outcome - equivocal |
| Importance of surgeons opinion post operatively |  |  |
|  |  |  |
| Overall health | Overall health |  |
|  |  |  |
| Importance of foot in RA | Importance of foot in RA |  |
|  |  |  |
| Descriptions of pain | Pain |  |
| Pain preventing sleep |  |  |
| Pain with activity |  |  |
| Pain with rest |  |  |
|  |  |  |
| Positive attitude | Positivity |  |
|  |  |  |
| Motivation of preventing future deformity | Motivation for surgery | Motivation for surgery - others opinion |
| Motivation to improve general health |  | Motivation for surgery - activity limitation |
| Motivation to reduce impact on other structures |  | Motivation for surgery - footwear |
| Motivation to prevent future pain |  | Motivation for surgery - preventing future damage/deformity |
| Motivation to help participation in valued activities |  |  |
|  |  |  |
| Patient's perception of optimum timing of surgery | Timing of surgery |  |
|  |  |  |
| Social support | Social support |  |
| Satisfaction with outcome |  |  |
| Negative experiences with surgeon | Surgeon related factors |  |
| Positive experiences with surgeon |  |  |
| Surgeons role prior to operation |  |  |
| Surgeons opinion of outcome |  |  |
|  |  |  |
| Patients wouldn’t have operation again | Whether patients would repeat surgery |  |
| Patients would have operation again |  |  |
